# Briacavatolides D–F, New Briaranes from the Taiwanese Octocoral *Briareum excavatum*

**DOI:** 10.3390/md10092103

**Published:** 2012-09-24

**Authors:** Shang-Kwei Wang, Tsun-Tai Yeh, Chang-Yih Duh

**Affiliations:** 1 Asia-Pacific Ocean Research Center, National Sun Yat-Sen University, Kaohsiung 804, Taiwan; Email: skwang@cc.kmu.edu.tw; 2 Department of Microbiology, Kaohsiung Medical University, Kaohsiung 807, Taiwan; 3 Department of Marine Biotechnology and Resources, National Sun Yat-Sen University, Kaohsiung 804, Taiwan; Email: m985020027@student.nsysu.edu.tw

**Keywords:** *Briareum excavatum*, briarane-type diterpenoid, cytotoxicity, anti-HCMV

## Abstract

In the continued search for novel bioactive substances from the Taiwanese octocoral *Briareum excavatum* collected at Orchid Island, three new briarane-type diterpenoids, briacavatolides D–F (**1**–**3**) were isolated from the acetone extract. The structures of these compounds were elucidated by extensive NMR spectroscopic analysis and physical data. The anti-HCMV (human cytomegalovirus) activity of **1**–**3** and their cytotoxicity against selected cancer cell lines were evaluated.

## 1. Introduction

Briarane-type diterpenoids, a group of diterpenoids having a highly oxidized bicyclo[8.4.0] system with a γ-lactone group are found only in marine organisms and mainly from octocorals [1,2,3,4]. The compounds of this type are proven to possess various bioactivities such as anti-inflammatory, cytotoxicity, and antibacterial activity [1,2,3,4]. Octocorals belonging to the genus *Briareum* (family Briareidae) are recognized as rich sources of briarane-type diterpenoids (3,8-cyclized cembranoid) [1,2,3]. 

**Figure 1:**
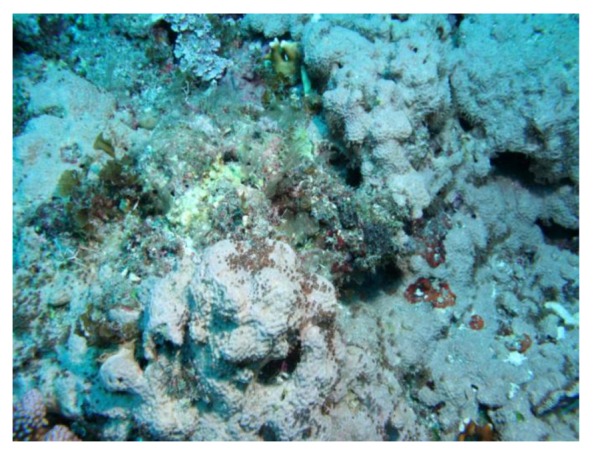
Octocoral* Briareum excavatum*.

**Figure 2:**
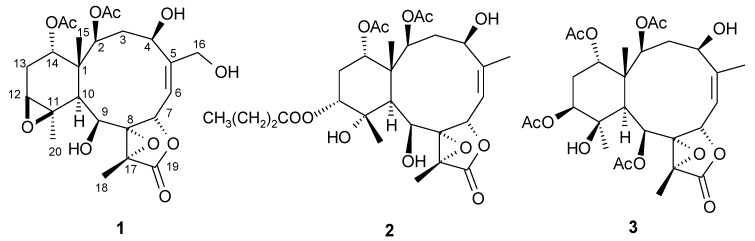
Structures of compounds **1–3**.

Human cytomegalovirus (HCMV) is a highly ubiquitous pathogen with a global prevalence of 60%~90% in the human population. For healthy people, HCMV remains a long-term subclinical infection, however, in congenital neonates and in immunocompromised patients the virus can cause severe diseases. Of the FDA approved therapeutic drugs, ganciclovir, foscarnet, and cidofovir are reported to have adverse effects on bone marrow and the kidneys. The continued chemical investigation of octocoral *B. excavatum *(Figure 1) collected at Orchid Island off Taiwan during August 2008 [5] afforded three new briarane-type diterpenoids, briacavatolides D–F (**1**–**3**) (Figure 2) (not to be confused with the related briaexcavatolides). The anti-HCMV (human cytomegalovirus) activity of **1**–**3** and their cytotoxicity against selected cell lines were evaluated.

## 2. Results and Discussion

The sliced bodies of the Taiwanese octocoral *B. excavatum* were exhaustively extracted with acetone. The combined acetone extracts were concentrated to a brown gum, which was further partitioned between H_2_O and EtOAc. The EtOAc-soluble portion was concentrated under reduced pressure and the residue was fractionated over silica gel 60 by column chromatography. The resulting fractions were further purified by RP-18 HPLC to yield the new compounds **1**–**3** (Figure 2).

**Figure 3:**
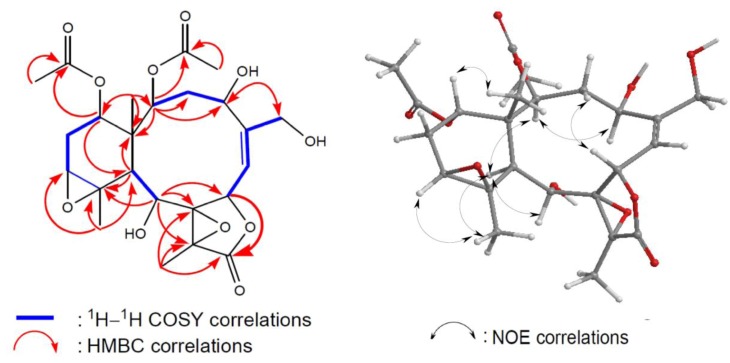
2D NMR correlations of compound **1**.

Briacavatolide D (**1**) was obtained as a white powder. Its HRMS and NMR spectral data established a molecular formula of C_24_H_32_O_11_, implying the existence of eight double bond equivalents. The ^1^H and ^13^C NMR spectra (Table 1) of **1** indicated the presence of two acetoxyls (*δ*_H_ 1.97, 2.03; *δ*_C_ 21.2, 21.1, 171.8, 170.6), a γ-lactone (*δ*_H_ 6.22; *δ*_C_ 172.2), and a trisubstituted olefin (*δ*_H_ 5.71; *δ*_C_ 149.3, 124.0). A tetrasubstituted epoxide containing a methyl substituent was indicateded by the signals of two quaternary oxygenated carbons (*δ*_C_ 62.6, 71.1) and a methyl (*δ*_C_ 9.3; *δ*_H_ 1.67, 3H, s). A trisubstituted epoxide containing a methyl substituent was revealed from the signals of an oxymethine (*δ*_C_ 61.4; *δ*_H_ 3.05) and a quaternary oxygenated carbons (*δ*_C_ 63.5) and a methyl (*δ*_C_ 24.6; *δ*_H_ 1.33, 3H, s). From the above data, metabolite **1** was found to be a pentacyclic compound. The structure and the ^1^H and ^13^C chemical shifts of **1 **were assigned by the assistance of 2D NMR studies, including ^1^H–^1^H COSY and HMBC experiments (Figure 3). By analysis of ^1^H–^1^H COSY correlations (Figure 3), it was possible to establish four partial structures of consecutive proton systems extending from H-2 to H-4; H_3_-16 to H-7 through H-6; H-6 to H-7; H-9 to H-10; and H-12 to H-14 through H-13. HMBC correlations (Figure 3) further led to the connectivities of the gross structure. According to the above observations, metabolite **1 **seemed to be very similar to 16-hydroxystecholide C acetate [6], which was previously isolated from the soft coral *Solenopodium excavatum*. By means of 1D and 2D NMR data it was found that the acetoxy groups at the C-4 and C-9 positions in 16-hydroxystecholide C acetate were replaced by hydroxy groups **1**. On the basis of the above finding, and by the NOE correlations of **1 **(Figure 3), bricavatolide D (**1**) was found to be the 4-*O*-deacetyl-9-*O*-deacetyl derivative of 16-hydroxystecholide C acetate.

Briacavatolide E (**2**) was isolated as a white solid and had the molecular formula C_28_H_40_O_12_, as determined by HRESIMS. The presence of hydroxyl, γ-lactone, and ester groups were evident from IR absorptions at 3447, 1773, and 1734 cm^−1^, respectively. ^13^C NMR spectral data (Table 1) revealed that **2** contains a trisubstituted double bond (*δ*_C_ 145.2, s, C-5; 122.0, d, C-6) and four carbonyl resonances (*δ*_C_ 171.0, 170.4, 170.2, and 172.5). Three esters were identified as two acetates and one *n*-butyrate by the presence of resonances in the ^1^H NMR spectrum of **2** at *δ*_H_ 1.97 (3H, s), 1.96 (3H, s), 0.96 (3H, t), 1.60 (1H, m) and 2.27 (H, m) (Table 1). 

**Figure 4:**
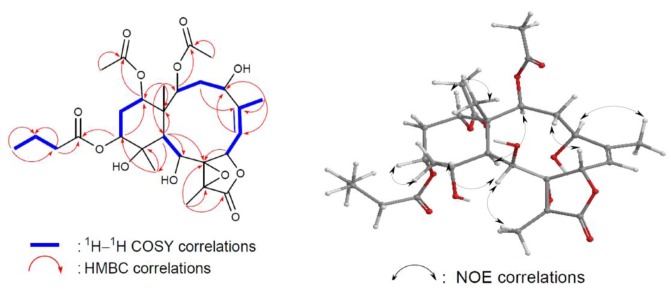
2D NMR correlations of compound **2**.

The gross structure of **2** and all of the ^1^H and ^13^C chemical shifts associated with the molecule were determined by a series of 2D NMR experiments (Figure 4). In the HMBC spectrum of **2**, the *n*-butyrate positioned at C-12 was confirmed from the long-range coupling between H-12 (*δ*_H_ 4.82) with the carbonyl carbon (*δ*_C_ 171.0) of the *n*-butyryloxyl group. Furthermore, the HMBC correlations also revealed that two acetates were attached to C-2 and C-14. These data, together with the other ^1^H-^13^C long-range correlations (Table 1), unambiguously established the molecular framework of 2. The relative configurations of **2** were identical to those of briaexcavatin L except that of C-12. H_3_-20 were found to exhibit NOE correlations (Figure 4) with H-12 and H-9, revealing the β-orientation of H-12 [7]. Splitting patterns of H-12 and H-14 as triplets (3.0 Hz) indicated that both are β equatorial.

**Table 1:** NMR spectroscopic data of **1**-**3**.

Position	1		2		3	
	*δ*_H_ (*J* in Hz) *^a^*	*δ*_C_ *^b^*	*δ*_H_ (*J* in Hz) *^a^*	*δ*_C_ *^b^*	*δ*_H_ (*J* in Hz) *^a^*	*δ*_C_ *^b^*
1		45.6		47.5		46.4
2	4.65 d (6.8) *^c^*	74.5	5.00 d (8.8)	74.9	4.86 d (7.6)	74.1
3	1.95 m	40.5	1.89 m	39.7	2.03 m	40.8
	3.17 dd (15.6, 12.8)		3.38 dd (15.0, 12.8)		2.92 dd (15.6, 12.0)	
4	4.34 dd (12.8, 4.8)	68.0	4.09 dd (12.0, 5.2)	71.2	4.21 dd (12.0, 5.6)	71.0
5		149.3		145.2		147.4
6	5.71 d (9.6)	124.0	5.35 d (9.0)	122.0	5.40 d (9.6)	121.5
7	6.22 d (9.6)	73.4	6.19 d (9.0)	75.3	6.00 d (9.6)	73.7
8		71.1		71.9		70.3
9	4.43 br s	71.6	4.73 d (3.6)	64.9	5.88 d (2.4)	67.5
10	2.21 d (3.6)	42.5	2.14 s	45.7	2.19 br s	47.8
11		63.5		74.1		75.5
12	3.05 d (5.2)	61.4	4.82 t (3.0)	73.9	4.91 dd (12.0, 5.2)	73.0
13	2.10 m	25.3	2.07 m 2.19 m	25.8	1.92 m 1.99 m	25.6
14	4.75 br s	73.7	4.66 t (3.0)	73.6	4.88 t (3.0)	75.7
15	1.20 s	16.0	1.34 s	14.2	1.34 s	15.8
16	4.31 br s	67.1	2.05 d (0.8)	25.4	2.12 d (1.6)	25.2
17		62.6		64.9		64.4
18	1.67 s	9.3	1.65 s	9.3	1.74 s	10.3
19		172.2		172.5		170.9
20	1.33 s	24.6	1.42 s	23.5	1.24 s	27.8
OAc	2.03 s	21.2	1.97 s	21.5	2.00 s	21.2
	1.97 s	21.1	1.96 s	21.3	2.07 s	21.5
		171.8		170.4	2.22 s	21.1
		170.6		170.2	2.04 s	21.3
						170.5
						168.1
						169.6
						170.4
OCOPr			0.96 t (7.2)	13.7		
			1.60 m	18.3		
			2.27 m	36.3		
				171.0		

*^a^* 400 MHz in CDCl_3_ (assigned by COSY, HSQC, and HMBC experiments); *^b^* 100 MHz in CDCl_3_ (assigned by DEPT, COSY, HSQC, and HMBC experiments); *^c^ J* values (Hz) in parentheses.

**Figure 5:**
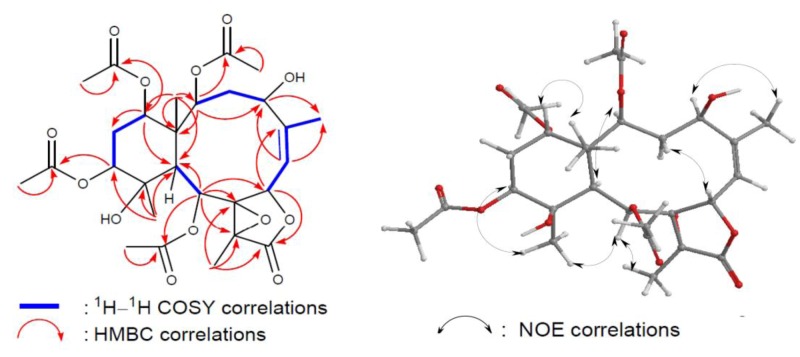
2D NMR correlations of compound **3**.

Briacavatolide F (**3**) had a molecular formula of C_28_H_38_O_13_ as deduced by HRESIMS. The IR spectrum of **3** indicated the presence of hydroxy (3501 cm^−1^), γ-lactone (1778 cm^−1^), and ester (1736 cm^−1^) groups. From the ^13^C NMR data of **3** (Table 1), a trisubstituted olefin (*δ* 147.4, C_q_, C-5; 121.5, CH, C-6) and five carbonyl resonances (*δ* 170.5, 170.4, 169.6, 168.1, 170.9) were derived. Four esters were identified as acetates by the presence of four methyl resonances in the ^1^H NMR spectrum of **3** at *δ *2.22 (3H, s), 2.07 (3H, s), 2.04 (3H, s), and 2.00 (3H, s) (Table 1). The planar structure of **3** was determined by 2D NMR experiments (Figure 5). The coupling information in the ^1^H–^1^H COSY experiment of **3** enabled identification of the C-2/3/4, C-6/16 (by allylic coupling), C-6/7, C-9/10, and C-12/13/14 units. From these data, together with the results of an HMBC experiment of **3**, the molecular framework of **3** could be further established. The HMBC data also revealed that the acetate groups are attached at C-2, C-9, C-12, and C-14; thus, the remaining hydroxy groups should be positioned at C-4 and C-11. The relative configurations of **3** elucidated from the NOE correlations (Figure 5) were the same as those of briaexcavatin L except C-11. NOE correlations from H_3_-20 to H-10/H-12 and from H-10 to H-12 revealed a β-axial orientation of hydroxy at C-11 for **3** [7]. The dd splitting pattern of H-12 (12.0, 5.2 Hz) indicated that H-12 is α axial. The triplet splitting pattern of H-14 is (3.0 Hz) indicated that H-14 is an β equatorial position; this is confirmed by a cross signal with Me-15.

The cytotoxicity toward P-388 (mouse lymphocytic leukemia), HT-29 (human colon adenocarcinoma), A-549 (human lung epithelial carcinoma) tumor cells, and human embryonic lung (HEL) cells as well as anti-HCMV activity of briacavatolides D–F (**1**–**3**) are shown in Table 2. Compound **3** showed moderate anti-HCMV activity.

**Table 2:** Cytotoxicity and anti- human cytomegalovirus (HCMV) activity of **1**–**3**.

Compounds	ED_50_ (μM)				Anti-HCMV
	A549	HT-29	P-388	HEL	
**1**	>100	>100	>100	>100	>100
**2**	>100	>100	>100	>100	>100
**3**	>100	>100	>100	>100	22

## 3. Experimental Section

### 3.1. General Experimental Procedures

Optical rotations were determined with a JASCO P1020 digital polarimeter. UV and IR spectra were obtained on JASCO V-650 and JASCO FT/IR-4100 spectrophotometers, respectively. NMR spectra were recorded on a Varian MR 400 NMR spectrometer at 400 MHz for ^1^H and 100 MHz for ^13^C, respectively. ^1^H NMR chemical shifts are expressed in *δ *values referring to the solvent peak *δ*_H_ 7.27 for CDCl_3_, and coupling constants are expressed in Hz. ^13^C NMR chemical shifts are expressed in *δ *values referring to the solvent peak *δ*_C_ 77.0 for CDCl_3_. MS were recorded by a Bruker APEX II mass spectrometer. Calculated values for positively charged ions were calibrated. Silica gel 60 (Merck, Germany, 230–400 mesh) and LiChroprep RP-18 (Merck, 40–63 μm) were used for column chromatography. Precoated silica gel plates (Merck, Kieselgel 60 F_254_, 0.25 mm) and precoated RP-18 F_254s_ plates (Merck) were used for thin-layer chromatography (TLC) analysis. High-performance liquid chromatography (HPLC) was carried out using a Hitachi L-7100 pump equipped with a Hitachi L-7400 UV detector at 220 nm together with a semi-preparative reversed-phase column (Merck, Hibar LiChrospher RP-18e, 5 μm, 250 × 25 mm).

### 3.2. Biological Material

The octocoral *B. excavatum* was collected by hand using scuba at Orchid Island off Taiwan, in July 2008 at a depth of 12 m and stored in a freezer until extraction. The voucher specimen (LY-05) was identified by Prof. Chang-Feng Dai, National Taiwan University and deposited at the Department of Marine Biotechnology and Resources, National Sun Yat-Sen University, Taiwan.

### 3.3. Extraction and Isolation

A specimen of octocoral *B. excavatum *(1.5 kg) was minced and extracted with acetone (2 L × 5) at room temperature. The combined acetone extracts was then partitioned between H_2_O and EtOAc. The resulting EtOAc extract (30.5 g) was subjected to gravity silica gel 60 column chromatography (Si 60 CC) using *n*-hexane and *n*-hexane/EtOAc of increasing polarity, to give 20 fractions. Fraction 12 (3.0 g), eluted with *n*-hexane/EtOAc (1:10), was further subjected to Si 60 CC (*n*-hexane/EtOAc, 10:1) to give 9 subfractions. A subfraction 12-5 (360 mg) was separated by a RP-18 flash column (MeOH/H_2_O, 50:50 to 100% MeOH) to give 6 fractions. In turn, a subfraction 12-5-2, eluted with MeOH/H_2_O (65:35), was further purified by RP-18 HPLC (MeOH/H_2_O, 60:40) to afford **3 **(2.0 mg). Likewise, the subfraction 13-9 (62 mg), was separated by a RP-18 flash column (MeOH/H_2_O, 40:60 to 100% MeOH) to give 7 fractions. In turn, a subfraction 13-9-2, eluted with MeOH/H_2_O (55:45), was further purified by RP-18 HPLC (MeOH/H_2_O, 37:50) to afford **1 **(1.5 mg). The fraction 14 (0.14 g), eluted with EtOAc/MeOH (70:1), was further subjected to a RP-18 flash column (MeOH/H_2_O, 40:60 to 100% MeOH) to give 7 fractions. The subfraction 14-1, eluted with MeOH/H_2_O (40:60), was purified by RP-18 HPLC (MeOH/H_2_O, 37:63) to afford **2** (1.5 mg).

Briacavatolide D (**1**): White amorphous powder; [α]_D_^25^ −33.6 (*c *0.2, CHCl_3_); IR (neat) ν_max_ 3419, 2925, 1776, 1727, 1372, 1260, 1024 cm^−1^; ^1^H NMR (CDCl_3_, 400 MHz) and ^13^C NMR (CDCl_3_, 100 MHz) data in Table 2; HRESIMS *m/z *519.1839 [M + Na]^+^ (calcd for C_24_H_32_O_11_Na, 519.1842).

Briacavatolide E (**2**): White amorphous powder; [α]_D_^25^ +22.3 (*c *0.1, CHCl_3_); IR (neat) ν_max_ 3447, 2964, 1773, 1734, 1371, 1262, 1022 cm^−1^; ^1^H NMR (CDCl_3_, 400 MHz) and ^13^C NMR (CDCl_3_, 100 MHz) data in Table 2; HRESIMS *m/z *591.2420 [M + Na]^+^ (calcd for C_28_H_40_O_12_Na, 591.2417).

Briacavatolide F (**3**): White amorphous powder; [α]_D_^25^ −27.6 (*c *0.1, CHCl_3_); IR (neat) ν_max_ 3501, 2941, 1778, 1736, 1372, 1252, 1016 cm^−1^; ^1^H NMR (CDCl_3_, 400 MHz) and ^13^C NMR (CDCl_3_, 100 MHz) data in Table 2; HRESIMS *m/z *605.2214 [M + Na]^+^ (calcd for C_28_H_38_O_13_Na, 605.2210).

### 3.4. Cytotoxicity Assay

Cytotoxicity was determined on P-388 (mouse lymphocytic leukemia), HT-29 (human colon adenocarcinoma), and A-549 (human lung epithelial carcinoma) tumor cells using a modification of the MTT colorimetric method according to a previously described procedure [8,9,10]. The provision of the P-388 cell line was supported by J. M. Pezzuto, formerly of the Department of Medicinal Chemistry and Pharmacognosy, University of Illinois at Chicago. HT-29 and A-549 cell lines were purchased from the American Type Culture Collection. To measure the cytotoxic activities of tested compounds, five concentrations with three replications were performed on each cell line. Mithramycin was used as a positive control.

### 3.5. Anti-HCMV Assay

To determine the effects of natural products upon HCMV cytopathic effect (CPE), confluent human embryonic lung (HEL) cells grown in 24-well plates were incubated for 1 h in the presence or absence of various concentrations of tested natural products with three replications. Ganciclovir was used as a positive control. Then, cells were infected with HCMV at an input of 1000 pfu (plaque forming units) per well of a 24-well dish. Antiviral activity was expressed as IC_50_ (50% inhibitory concentration), or compound concentration required to reduce virus induced CPE by 50% after 7 days as compared with the untreated control. To monitor the cell growth upon treating with natural products, an MTT-colorimetric assay was employed [8,11].

## 4. Conclusion

The continued investigation of Taiwanese octocoral *B. excavatum* collected at Orchid Island has led to the isolation of three new briarane-type diterpenoids, briacavatolides D–F (**1**–**3**). Briacavatolide F (**3**) was found to show anti-HCMV activity with an IC_50_ of 22 μM. 

## Supplementary Files

Supplementary File 1: PDF-Document (PDF, 2256 KB)
