# Supplementary material for: Briacavatolides D–F, New Briaranes from the Taiwanese Octocoral Briareum excavatum
Source: Mar Drugs. 2012 Sep 24;10(9):2103–10. doi: 10.3390/md10092103 (PMC3475276; doi:10.3390/md10092103)

# Supplementary Information

**Table of Contents**

Figure S1. $^{1}$H NMR spectrum (400 MHz) of briacavatolide D (**1**) in $CDCl_3$. 3

Figure S2. $^{13}$C NMR spectrum (100 MHz) of briacavatolide D (**1**) in $CDCl_3$. 4

Figure S3. COSY spectrum (400 MHz) of briacavatolide D (**1**) in $CDCl_3$. 5

Figure S4. HSQC spectrum (400 MHz) of briacavatolide D (**1**) in $CDCl_3$ 6

Figure S5. HMBC spectrum (400 MHz) of briacavatolide D (**1**) in $CDCl_3$. 7

Figure S6. NOESY spectrum (400 MHz) of p briacavatolide D (**1**) in $CDCl_3$. 8

Figure S7. $^{1}$H NMR spectrum (400 MHz) of briacavatolide E (**2**) in $CDCl_3$. 9

Figure S8. $^{13}$C NMR spectrum (100 MHz) of briacavatolide E (**2**) in $CDCl_3$. 10

Figure S9. COSY spectrum (400 MHz) of briacavatolide E (**2**) in $CDCl_3$. 11

Figure S10. HSQC spectrum (400 MHz) of briacavatolide E (**2**) in $CDCl_3$ 12

Figure S11. HMBC spectrum (400 MHz) of briacavatolide E (**2**) in $CDCl_3$. 13

Figure S12. NOESY spectrum (400 MHz) of p briacavatolide E (**2**) in $CDCl_3$. 14

Figure S13. $^{1}$H NMR spectrum (400 MHz) of briacavatolide F (**3**) in $CDCl_3$. 15

Figure S14. $^{13}$C NMR spectrum (100 MHz) of briacavatolide F (**3**) in $CDCl_3$. 16

Figure S15. COSY spectrum (400 MHz) of briacavatolide F (**3**) in $CDCl_3$. 17

Figure S16. HSQC spectrum (400 MHz) of briacavatolide F (**3**) in $CDCl_3$ 18

Figure S17. HMBC spectrum (400 MHz) of briacavatolide F (**3**) in $CDCl_3$. 19

Figure S18. NOESY spectrum (400 MHz) of briacavatolide F (**3**) in $CDCl_3$. 20

**Figure S1.** $^{1}$H NMR spectrum (400 MHz) of briacavatolide D (**1**) in $CDCl_3$.

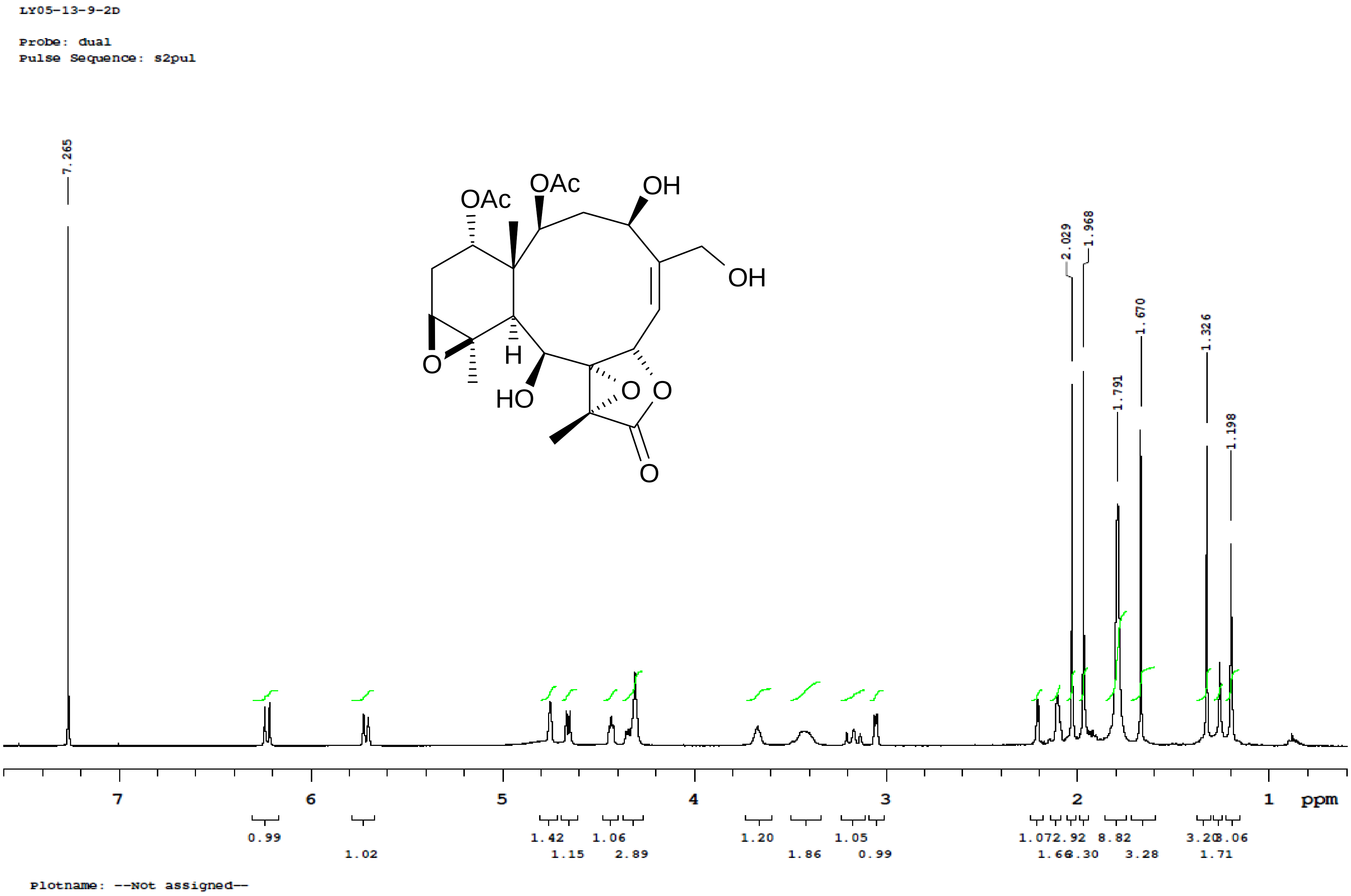

**Figure S2.** $^{13}C$ NMR spectrum (100 MHz) of briacavatolide D (**1**) in $CDCl_3$.

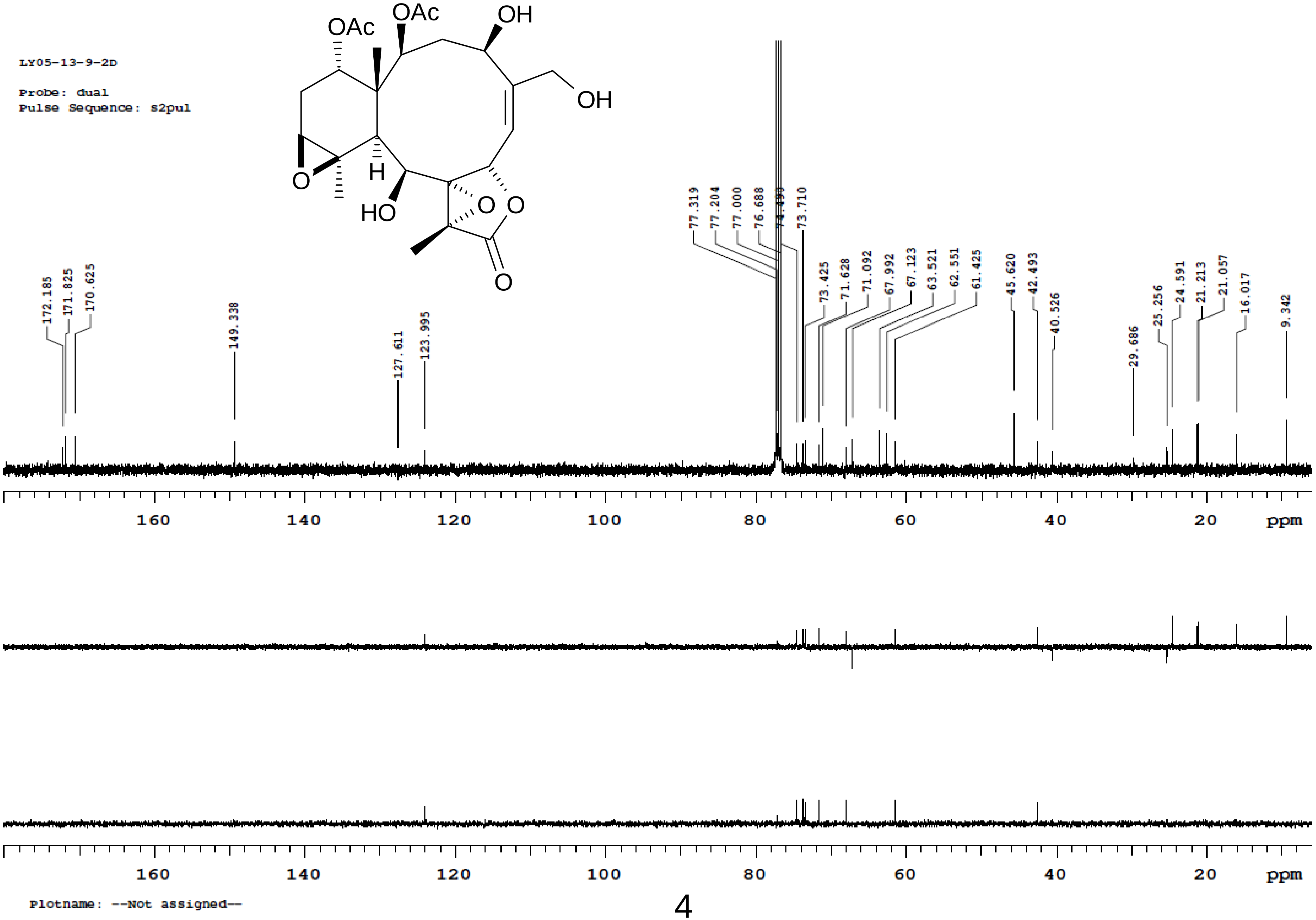

**Figure S3.** COSY spectrum (400 MHz) of briacavatolide D (**1**) in $CDCl_3$.

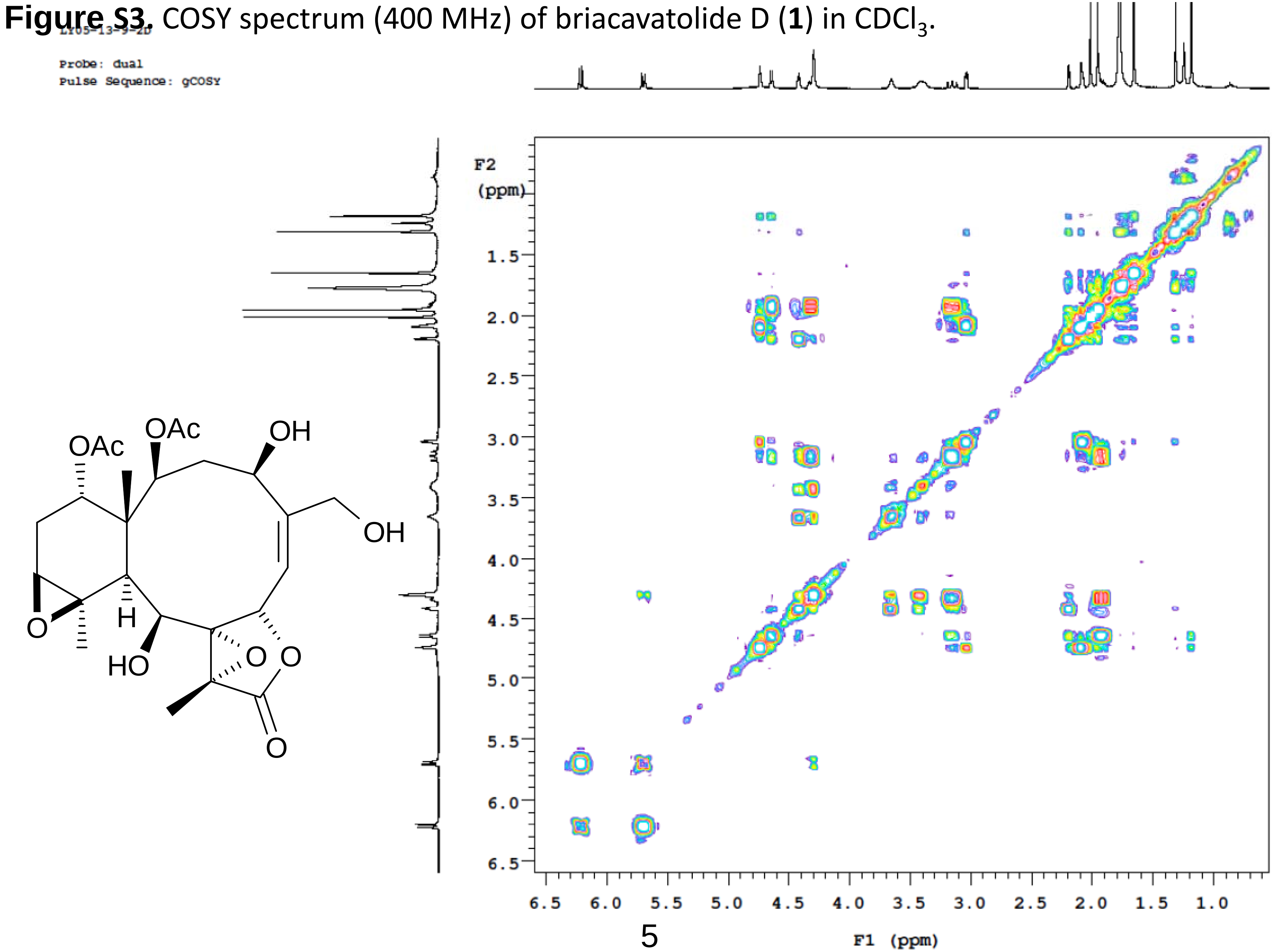

**Figure S4.** HSQC spectrum (400 MHz) of briacavatolide D (**1**) in $CDCl_3$

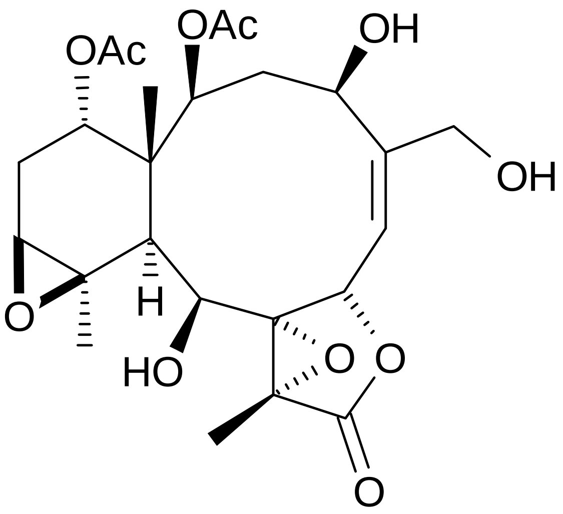


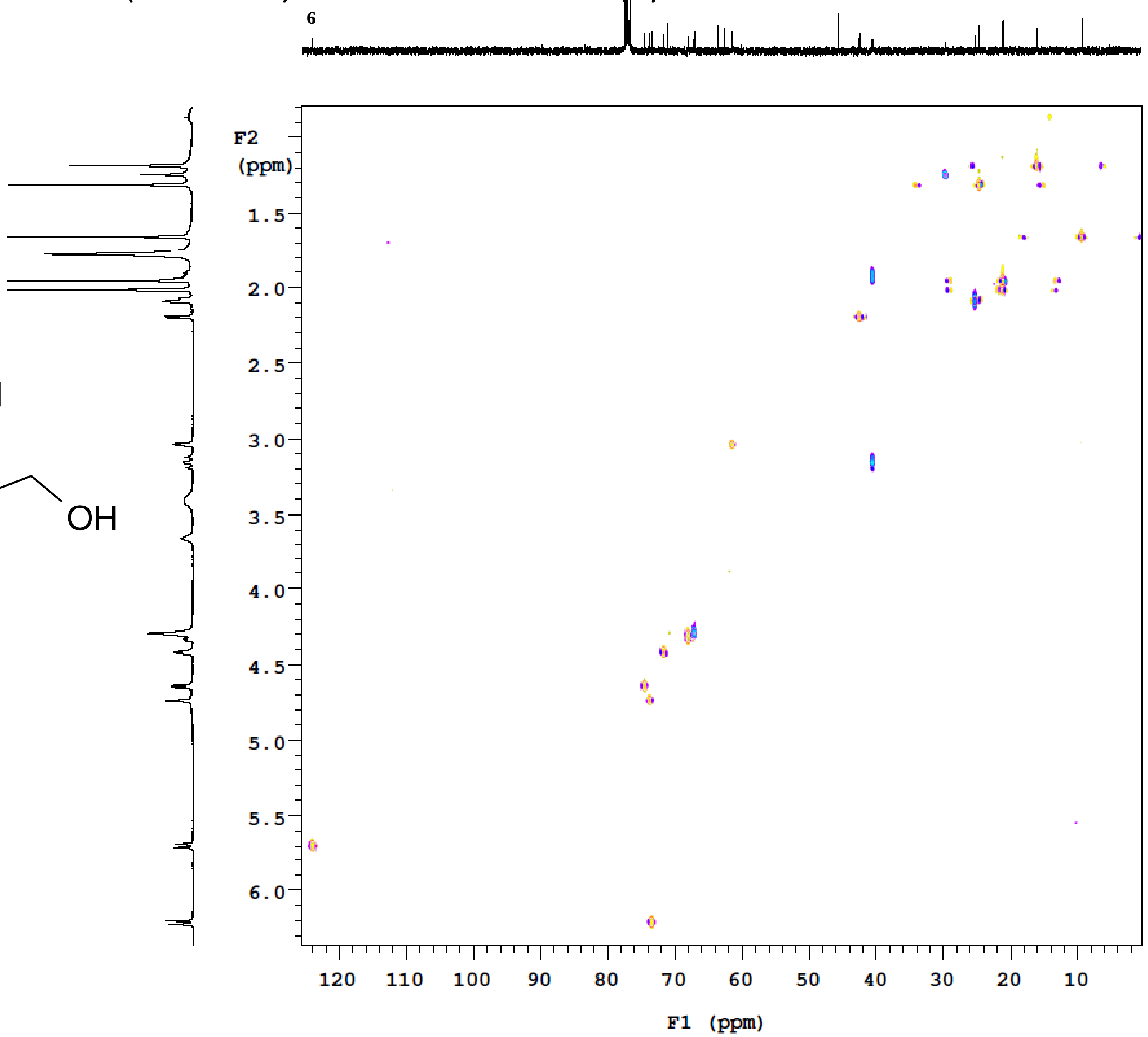

**Figure S5.** HMBC spectrum (400 MHz) of briacavatolide D (**1**) in $CDCl_3$.

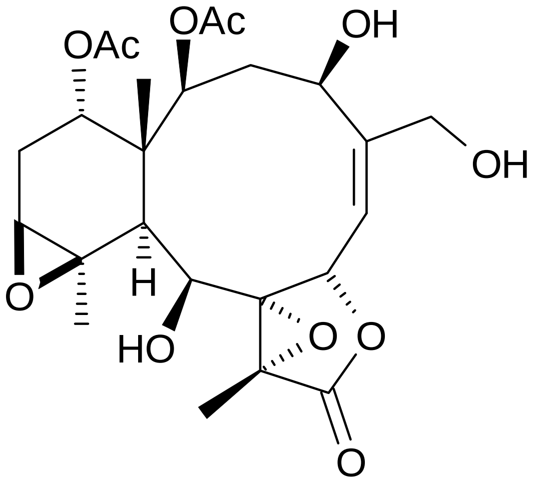


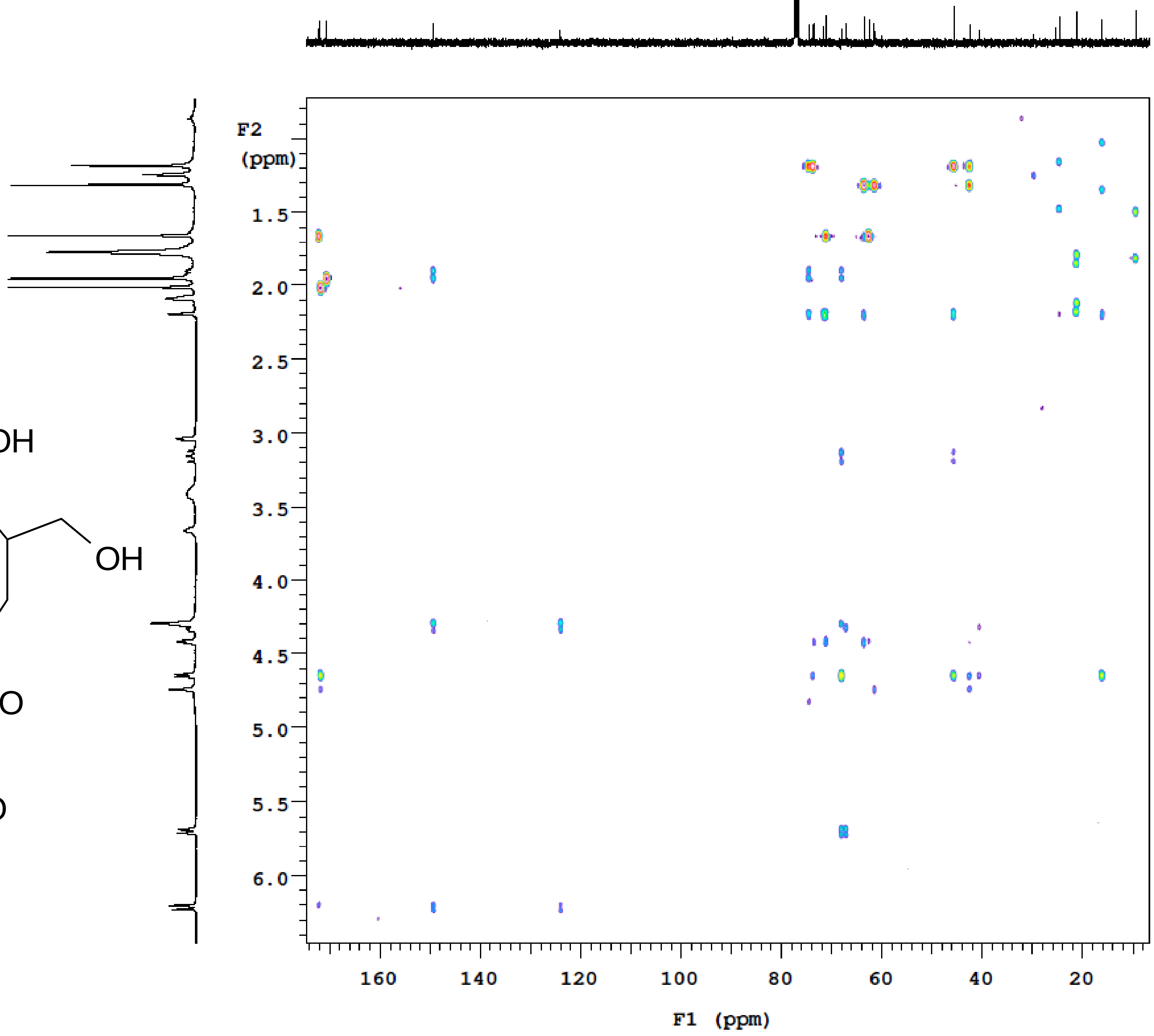

**Figure S6.** NOESY spectrum (400 MHz) of briacavatolide D (**1**) in $CDCl_3$.

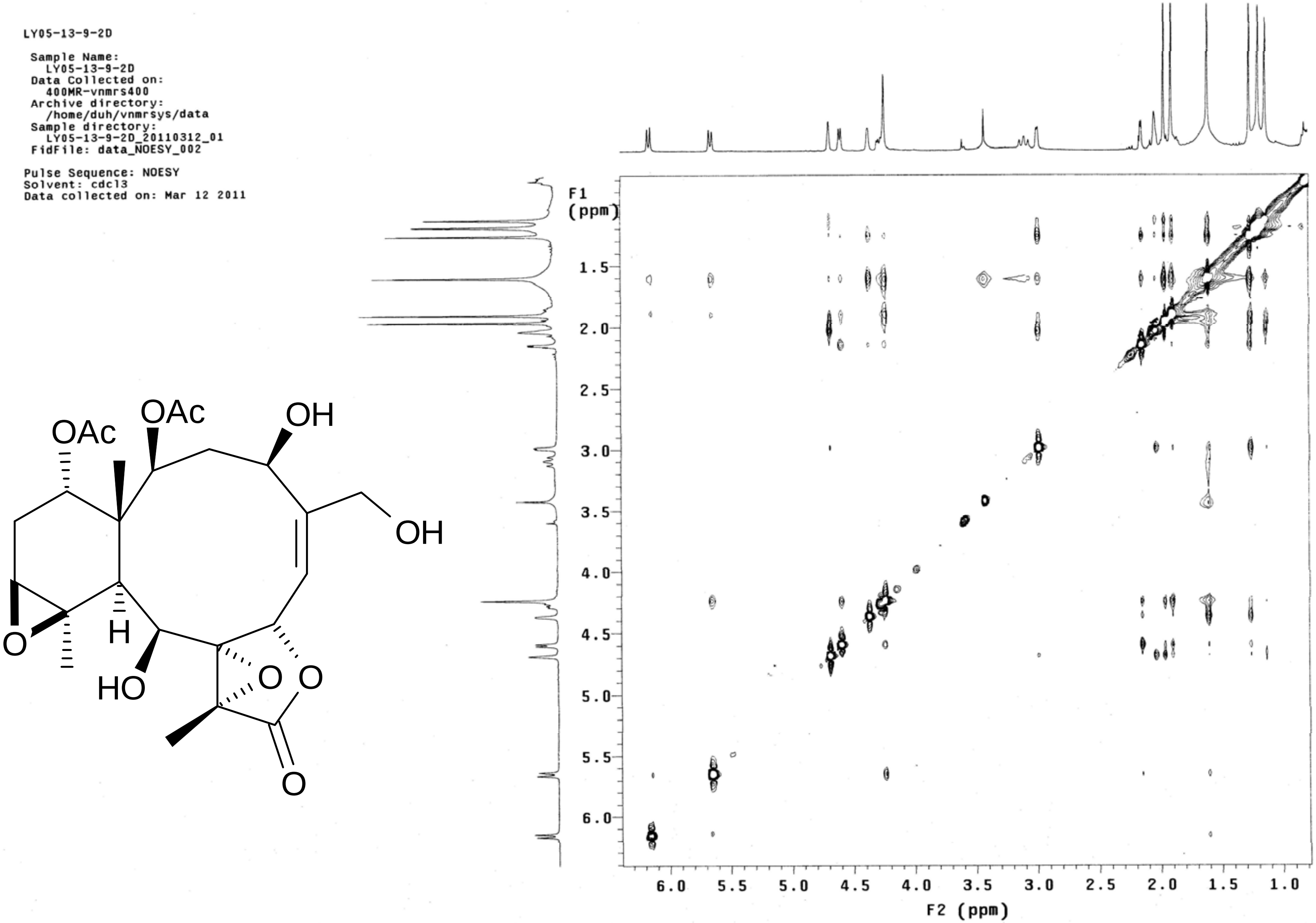

**Figure S7.** $^{13}C$ NMR spectrum (100 MHz) of briacavatolide E (**2**) in $CDCl_3$.

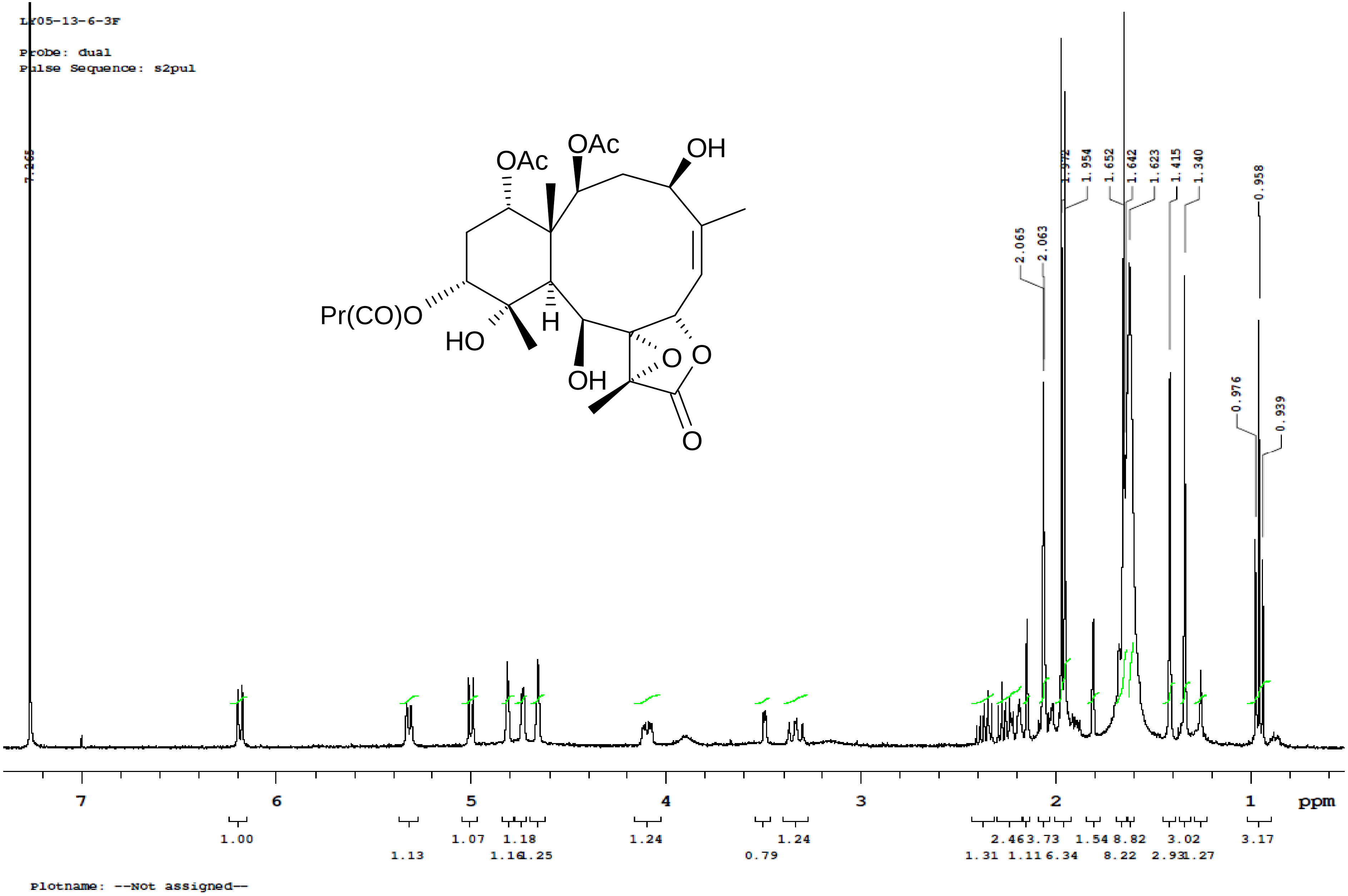

**Figure S8.** $^{13}$C NMR spectrum (100 MHz) of briacavatolide E (**2**) in $CDCl_3$.

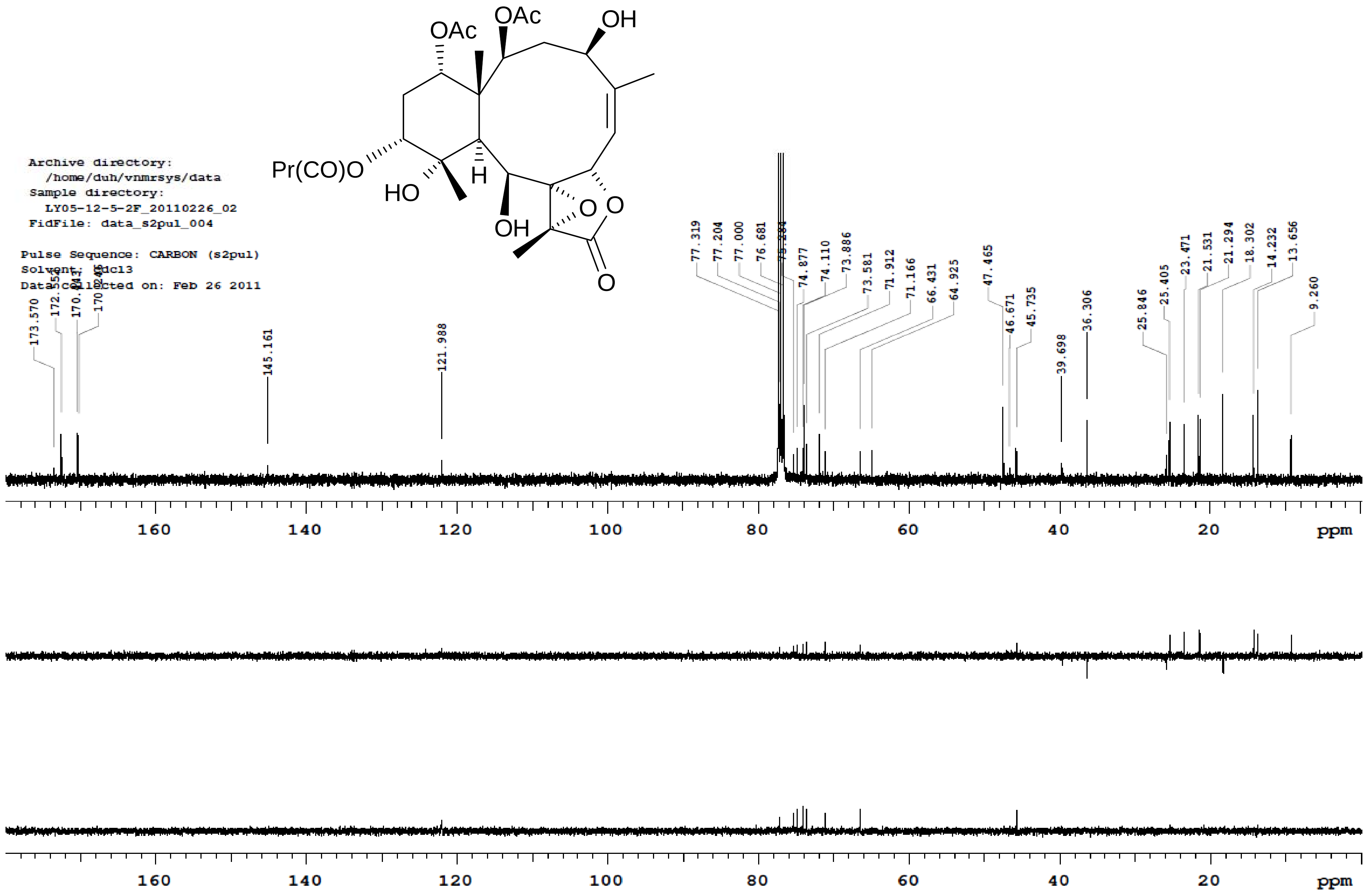

**Figure S9.** COSY spectrum (400 MHz) of briacavatolide E (**2**) in $CDCl_3$.

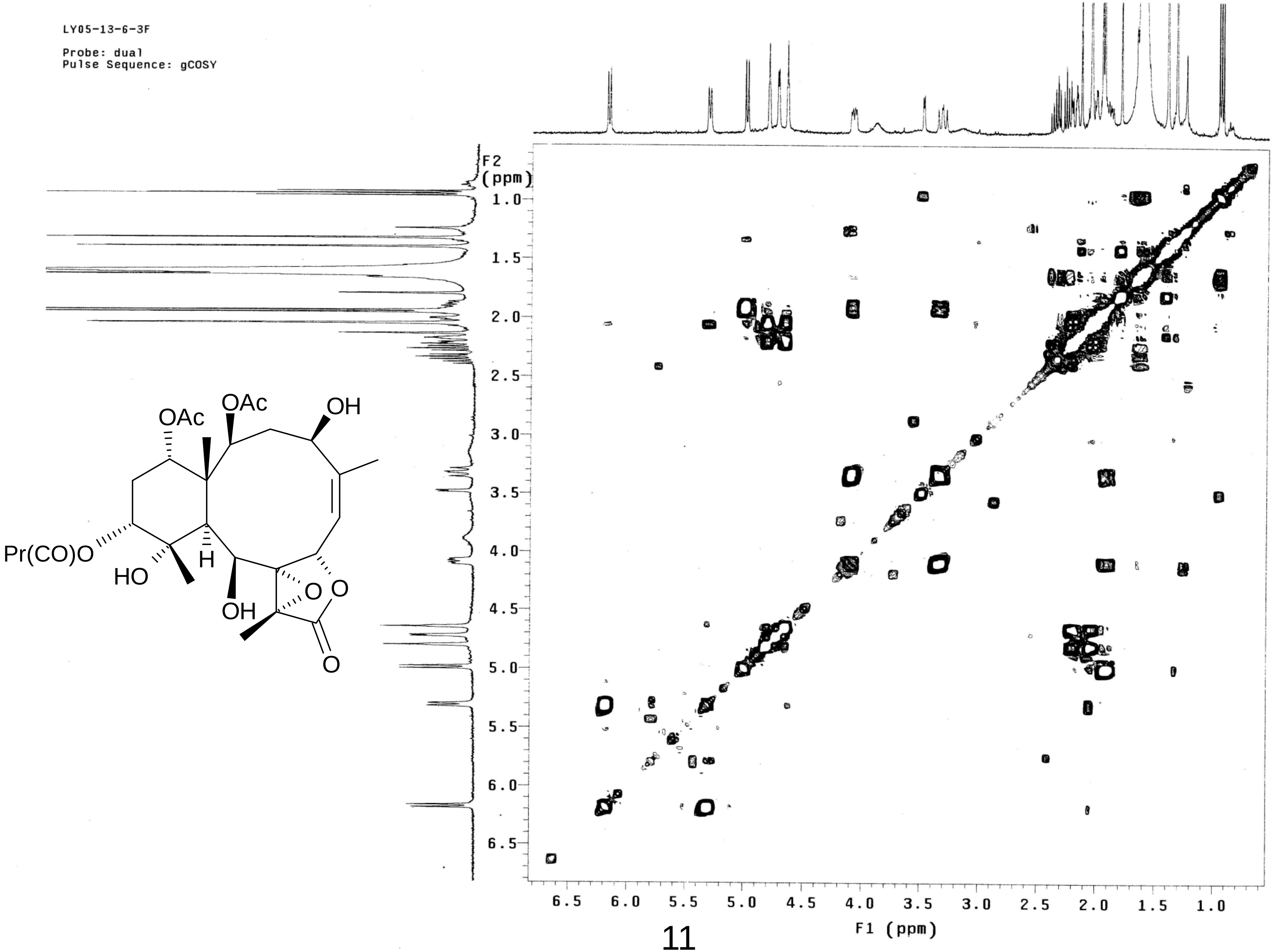

**Figure S10.** HSQC spectrum (400 MHz) of briacavatolide E (**2**) in $CDCl_3$.

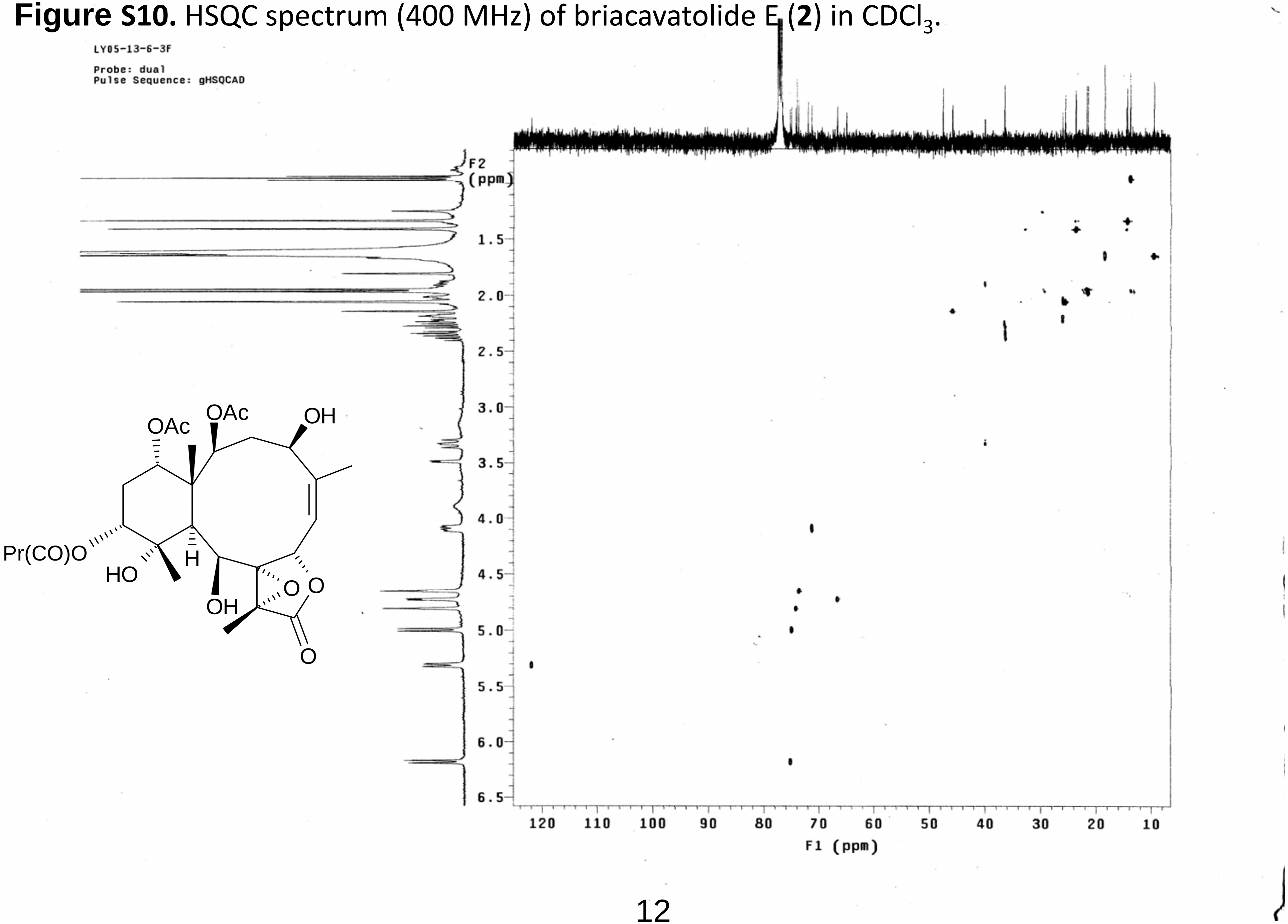

**Figure S11.** HMBC spectrum (400 MHz) of briacavatolide E (**2**) in $CDCl_3$.

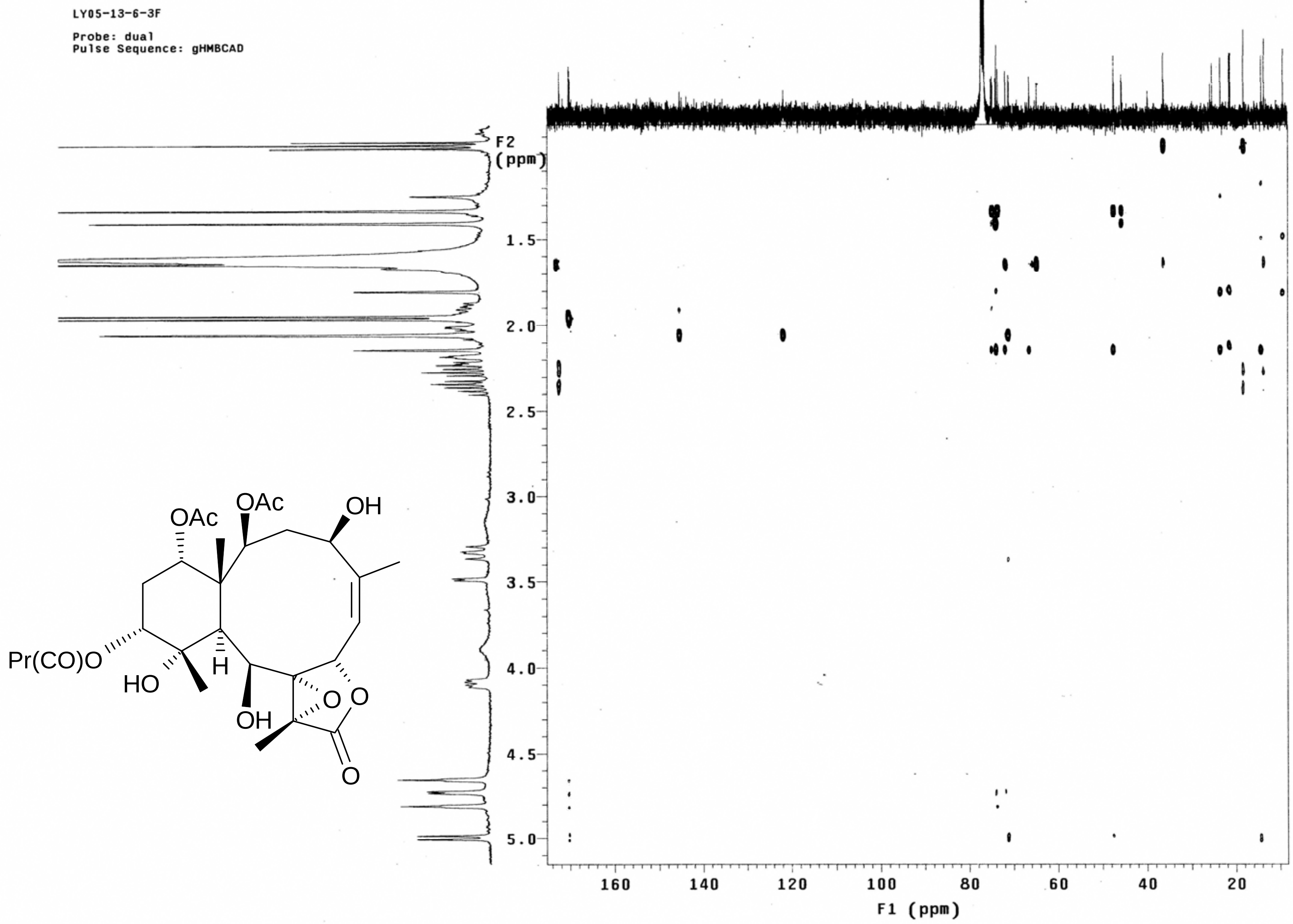

**Figure S12.** NOESY spectrum (400 MHz) of briacavatolide E (**2**) in $CDCl_3$.

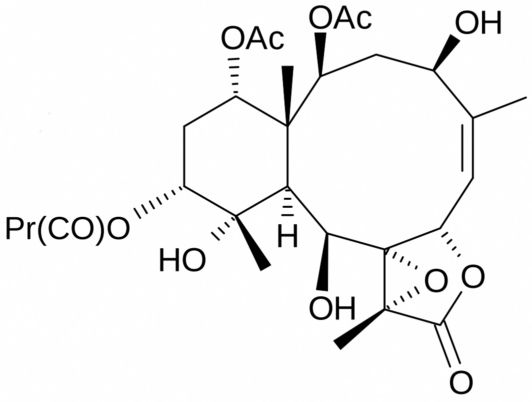


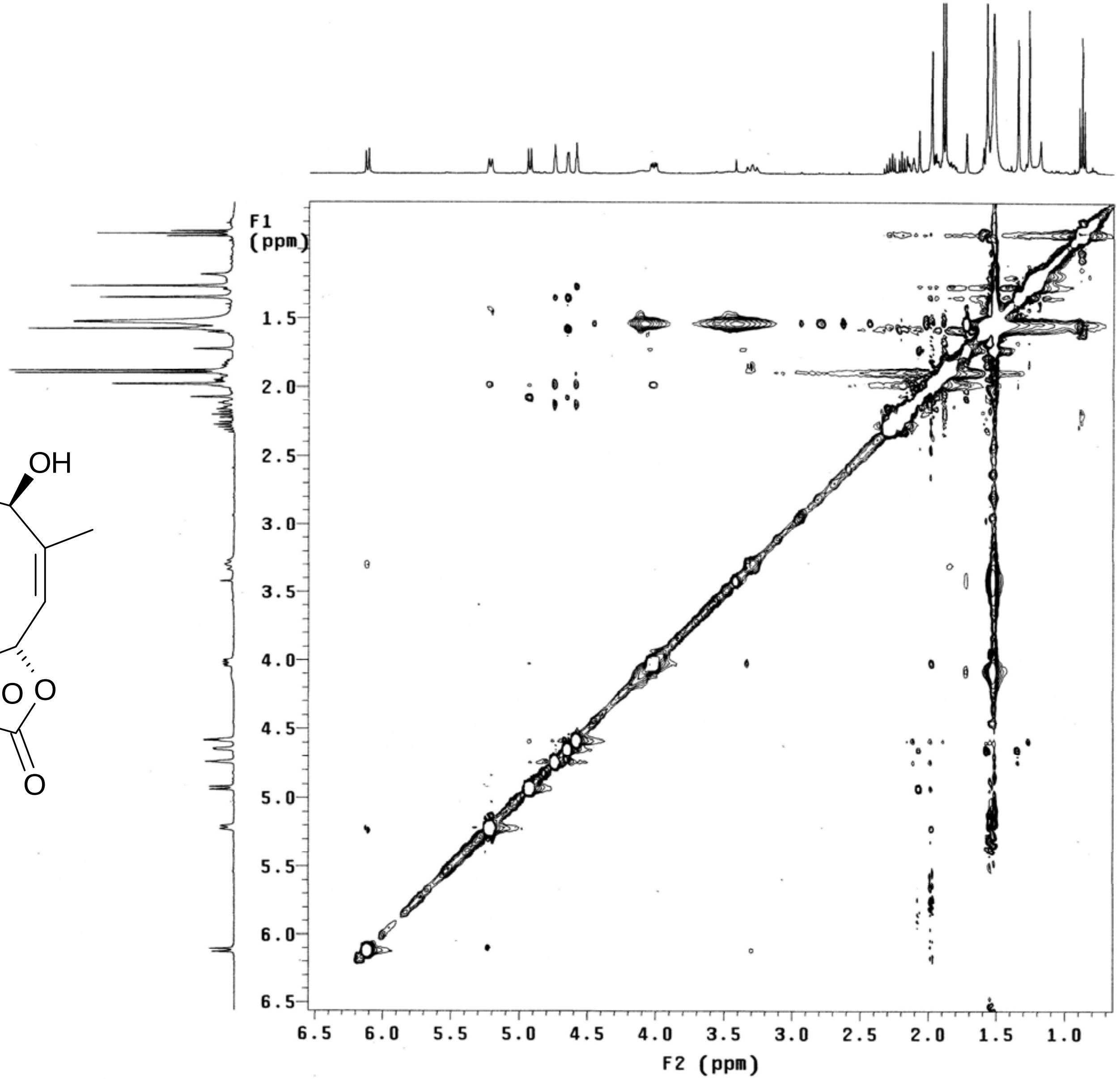

**Figure S13.** $^1$H NMR spectrum (400 MHz) of briacavatolide F (**3**) in $CDCl_3$.

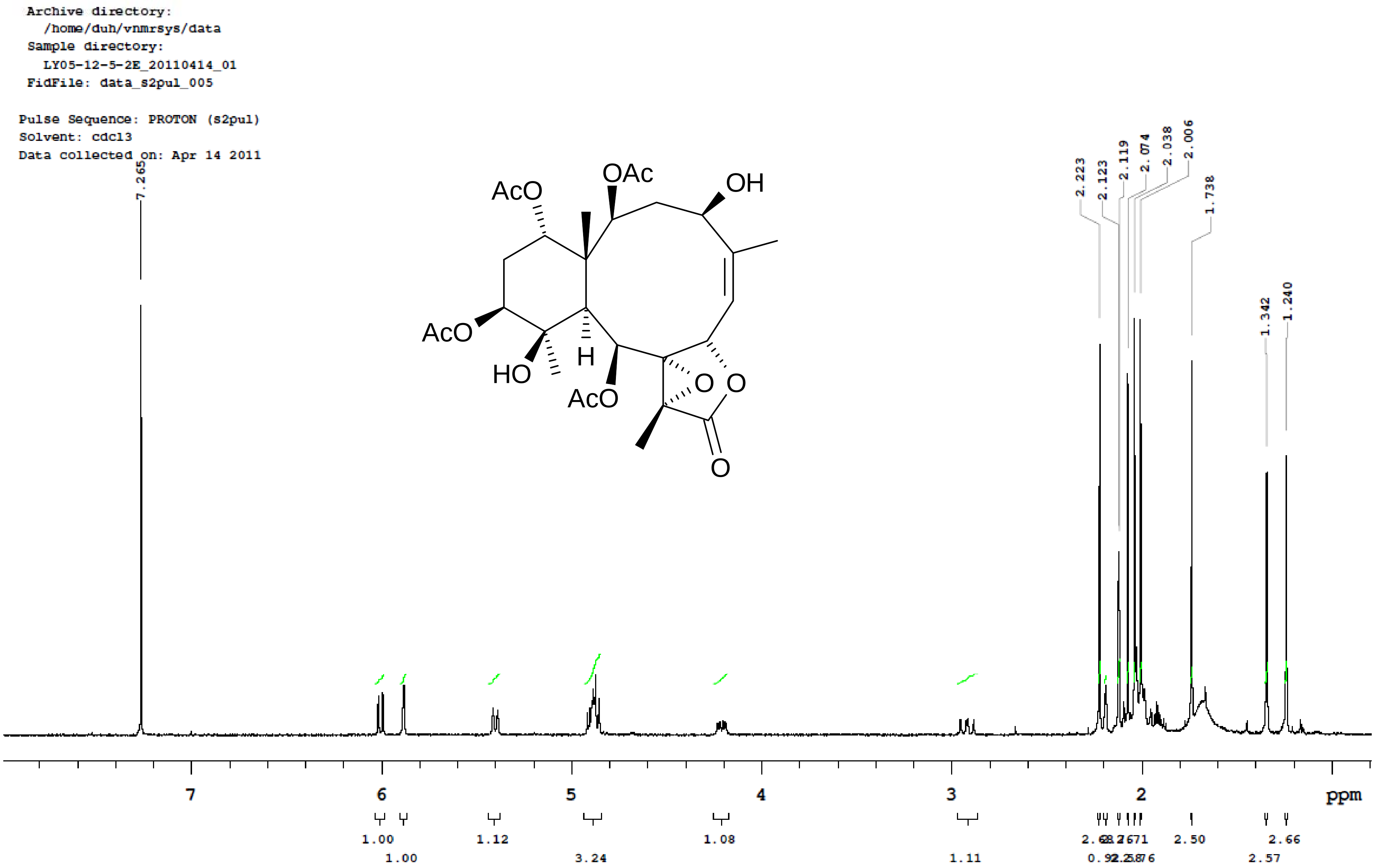

**Figure S14.** $^{13}$C NMR spectrum (100 MHz) of briacavatolide F (**3**) in $CDCl_3$.

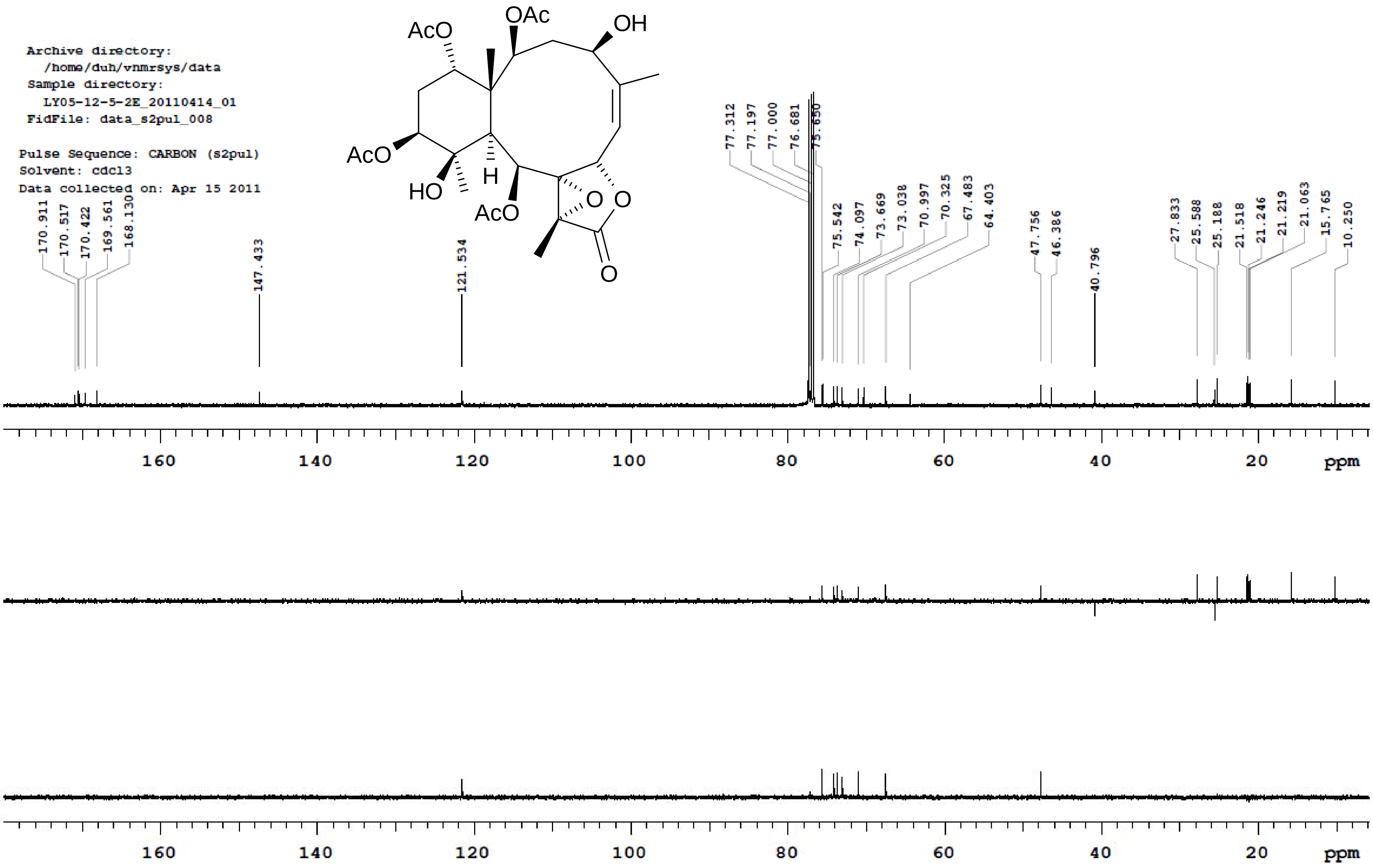

**Figure S15.** COSY spectrum (400 MHz) of briacavatolide F (**3**) in $CDCl_3$.

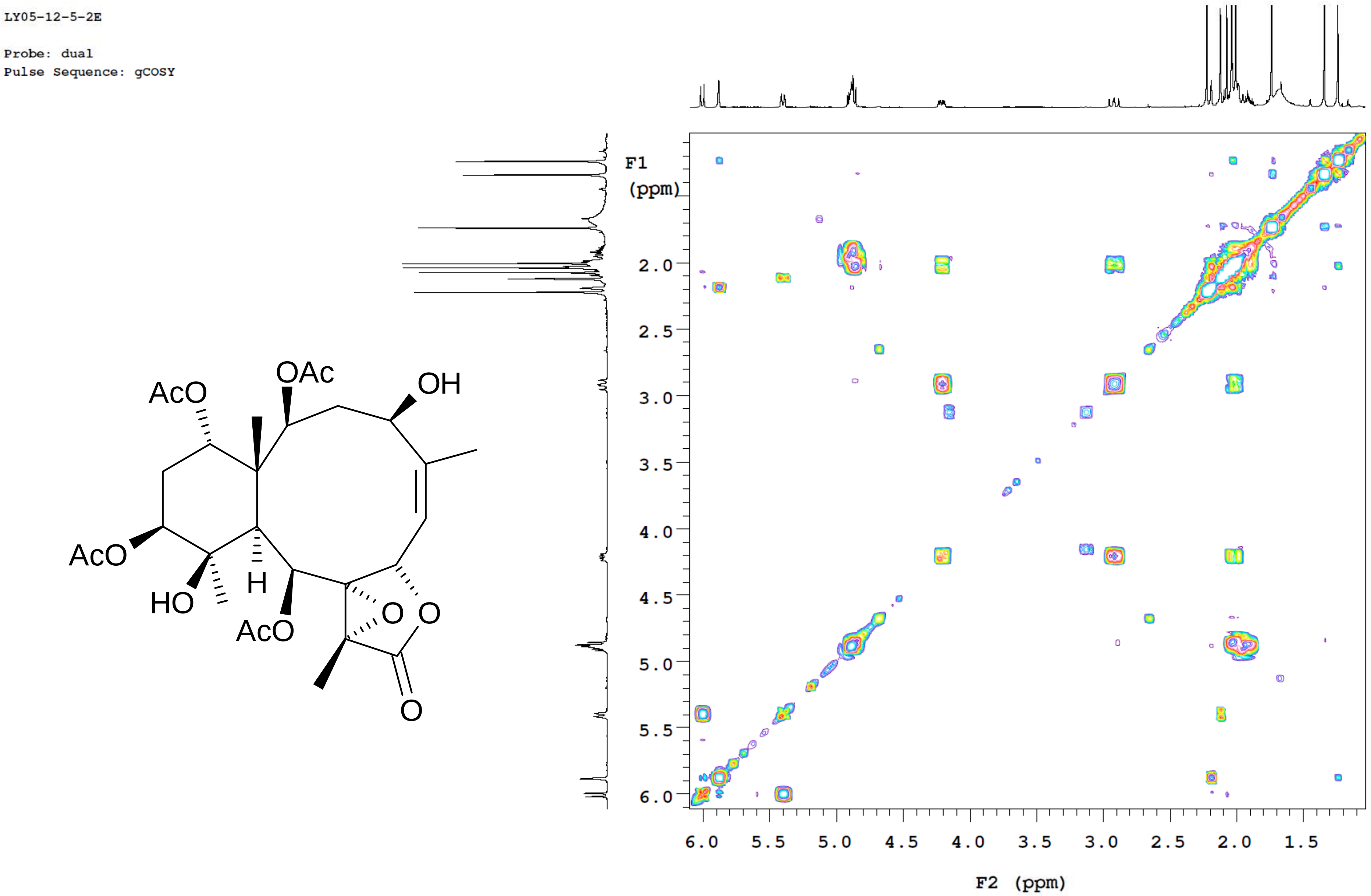

**Figure S16.** HSQC spectrum (400 MHz) of briacavatolide F (**3**) in $CDCl_3$.

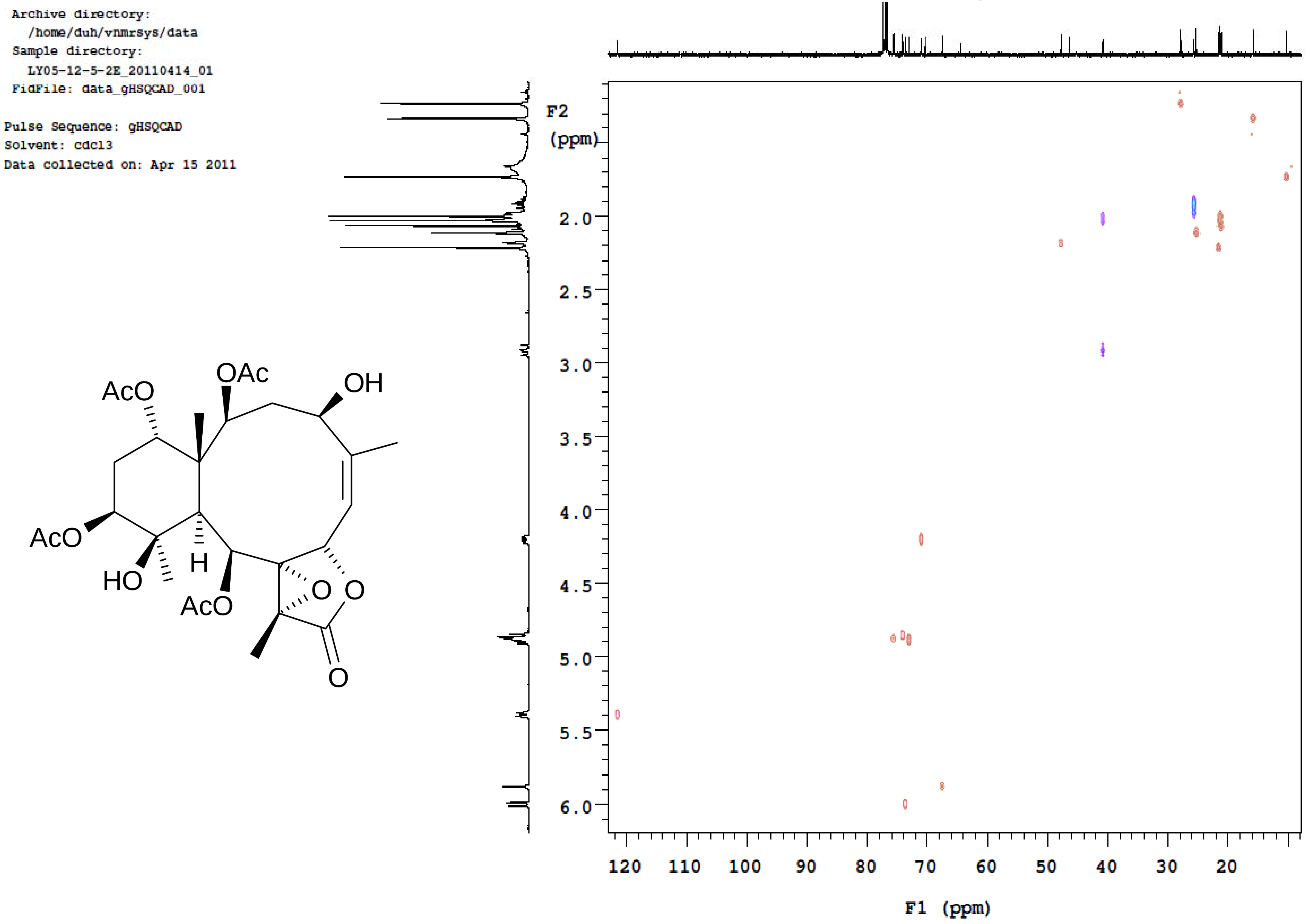

**Figure S17.** HMBC spectrum (400 MHz) of briacavatolide F (**3**) in $CDCl_3$.

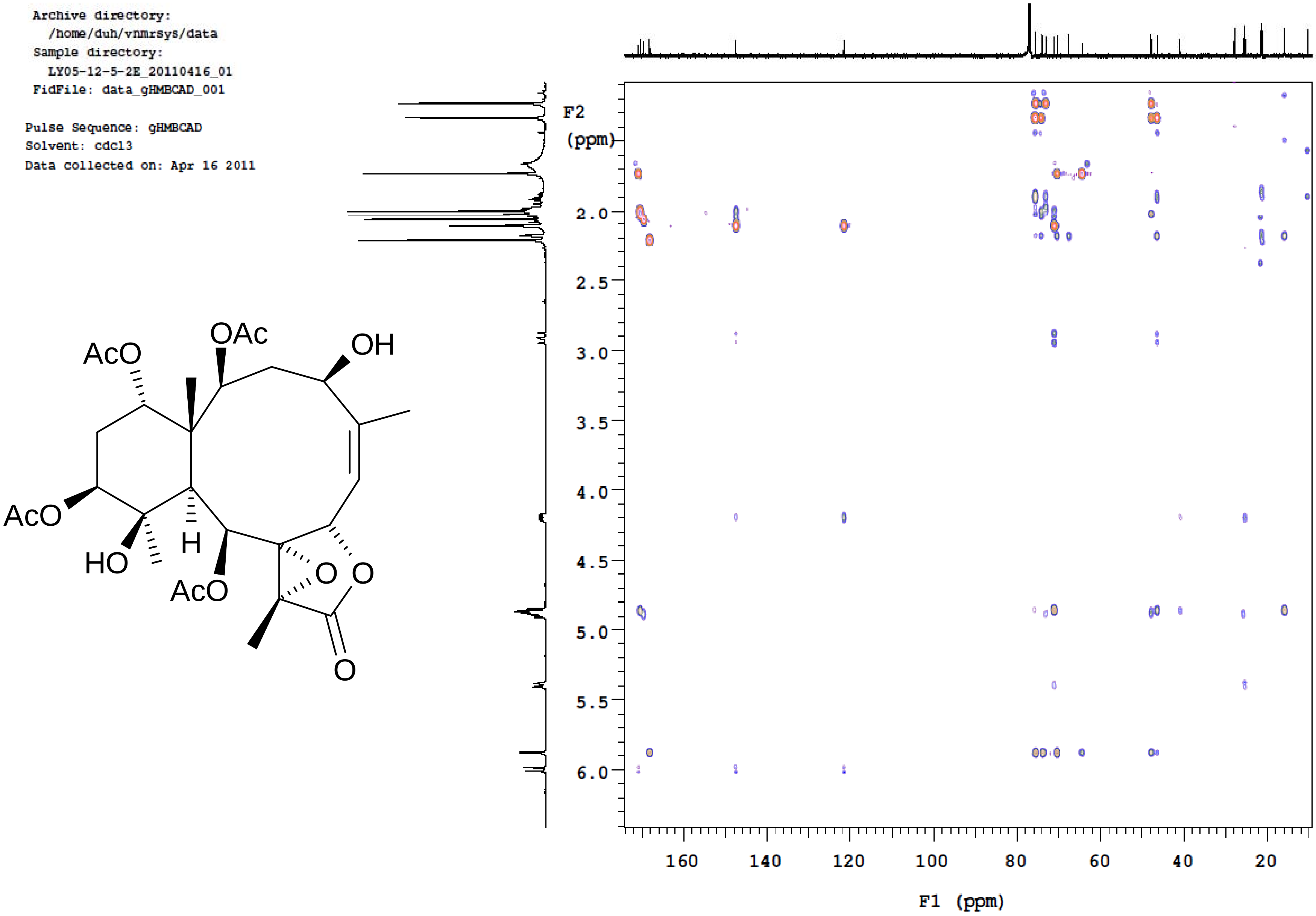

**Figure S18.** NOESY spectrum (400 MHz) of briacavatolide F (**3**) in $CDCl_3$.

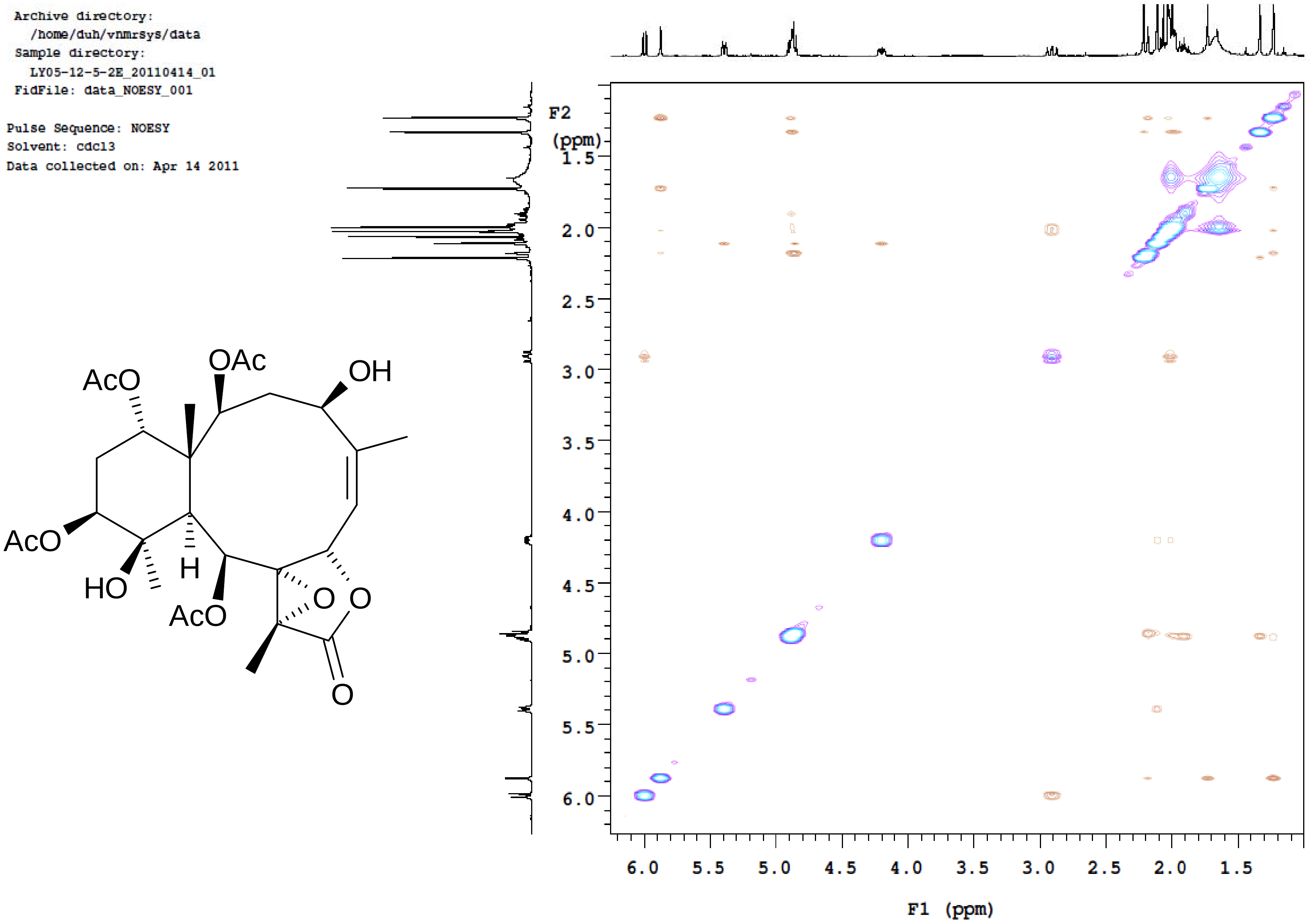

Supplement: Supplementary File 1: — PDF-Document (PDF, 2256 KB) [file marinedrugs-10-02103-s001.pdf]
